# STAT3 modulates *β*-cell cycling in injured mouse pancreas and protects against DNA damage

**DOI:** 10.1038/cddis.2016.171

**Published:** 2016-06-23

**Authors:** S De Groef, D Renmans, Y Cai, G Leuckx, S Roels, W Staels, G Gradwohl, L Baeyens, Y Heremans, G A Martens, N De Leu, M Sojoodi, M Van de Casteele, H Heimberg

**Affiliations:** 1 Diabetes Research Center, Vrije Universiteit Brussel, Laarbeeklaan 103, Brussels 1090, Belgium; 2 Division of Pediatric Endocrinology, Department of Pediatrics, Ghent University Hospital, Ghent, Belgium; 3 Department of Pediatrics and Genetics, Ghent University, Ghent, Belgium; 4 Development and Stem Cells, Institute of Genetics and Molecular and Cellular Biology (IGBMC), Illkirch 67404, France; 5 Diabetes Center, Eli and Edythe Broad Center for Regenerative Medicine and Stem Cell Research, University of California San Francisco, San Francisco, CA 94143-0669, USA; 6 Department of Clinical Chemistry and Radio-immunology, Universitair Ziekenhuis Brussel (UZ Brussel), Laarbeeklaan 101, Brussels 1090 Belgium; 7 Department of Endocrinology, UZ Brussel, Brussels 1090, Belgium; 8 ASZ Aalst, Aalst, Belgium

## Abstract

Partial pancreatic duct ligation (PDL) of mouse pancreas induces a doubling of the *β*-cell mass mainly through proliferation of pre-existing and newly formed *β*-cells. The molecular mechanism governing this process is still largely unknown. Given the inflammatory nature of PDL and inflammation-induced signaling via the signal transducer and activator of transcription 3 (STAT3), the activation and the role of STAT3 in PDL-induced *β*-cell proliferation were investigated. Duct ligation stimulates the expression of several cytokines that can act as ligands inducing STAT3 signaling and phosphorylation in *β*-cells. *β*-Cell cycling increased by conditional *β*-cell-specific *Stat3* knockout and decreased by STAT3 activation through administration of interleukin-6. In addition, the level of DNA damage in *β*-cells of PDL pancreas increased after deletion of *Stat3*. These data indicate a role for STAT3 in maintaining a steady state in the *β*-cell, by modulating its cell cycle and protection from DNA damage.

Signal transducers and activators of transcription (STAT) proteins are SH2 domain-containing cytoplasmic transcription factors expressed by various cell types. On receptor binding, specific cytokines, growth factors and hormones induce tyrosine phosphorylation, dimerization and activation of STAT proteins.^1^ Although discrete phenotypes have been described after ablation of most *Stat* genes, *Stat3* knockout results in early embryonic lethality.^2^ This ubiquitously expressed transcription factor is activated by a large number of ligands, including all members of the interleukin 6 (IL6) cytokine family. Among other factors that activate STAT3 in a cell-dependent manner are the IL10 cytokine family, interferons (IFNs), leptin, prolactin, epidermal growth factor (EGF), vascular endothelial growth factor (VEGF), Hepatocyte growth factor (HGF), platelet-derived growth factor (PDGF) and colony-stimulating factors (CSFs).^2, 3^ Tissue-specific conditional knockout of *Stat3* identified a role for STAT3 in cell migration,^4, 5, 6^ cell proliferation,^7^ (anti/pro)apoptotic signaling,^7, 8^ expression of acute-phase response genes,^9^ anti-inflammatory^10^ and neurotrophic signaling.^11^

In *Stat3*^lox/lox^ mice that express Cre recombinase under the control of the *Pdx1* promoter,^12, 13^ some studies excluded a role for STAT3 in the development and function of the pancreatic *β*-cells,^13^ whereas others observed glucose intolerance and impaired insulin secretion as a result of reduced VEGF-A signaling and abnormal islet vascularization.^12, 14^ *Stat3*^lox/lox^ mice with insulin promotor-driven Cre recombinase are glucose intolerant.^15, 16^ Although these studies suggest a contribution for STAT3 in *β*-cell function,^16^ its role in *β*-cell proliferation and survival is unknown. Mature *β*-cells mainly renew by self-replication^17, 18^ and their replication potential is homogenous and delayed by a postmitotic quiescence period under normal physiological conditions and following minor injury.^19, 20^ We have previously shown that severe pancreatic injury impacted by partial pancreatic duct ligation (PDL) leads to a doubling in *β*-cell mass^21^ that mainly results from proliferation of *β*-cells, prone to re-divide without refractory period.^22^ This process occurs distal to the ligation of the main pancreatic duct, suggesting autocrine or paracrine regulation, likely induced by the inflammatory environment of PDL that is characterized by expression of transforming growth factor-*α*, gastrin, IL1a, IL1b, IL6, IL10, IFN-*γ* and tumor necrosis factor.^23, 24, 25^ However, this list of factors is likely incomplete and their role in PDL-induced *β*-cell proliferation remains to be determined. Growth factors such as those of the IGF and EGF family, lactogens, hepatocyte growth factor (HGF), glucagon-like peptide-1 and parathyroid hormone-related protein stimulate rodent *β*-cell proliferation and survival *in vitro* and *in vivo.*^26, 27, 28^ In addition, cytokines, including members of the IL6 family, IL10 and IL27, regulate *β*-cell survival.^26, 27, 28, 29, 30, 31, 32, 33^ As some of these factors are able to activate STAT3, a role for STAT3 in *β*-cell mass maintenance can be envisioned.

We report activation of STAT3 in *β*-cells of PDL pancreas and identified factors that are potential activators of the JAK2/STAT3 pathway. In PDL pancreas, STAT3 modulates the *β*-cell cycle and protects *β*-cells from DNA damage.

## Results

### PDL-induced injury activates STAT3 in *β*-cells

**Figure 1:**
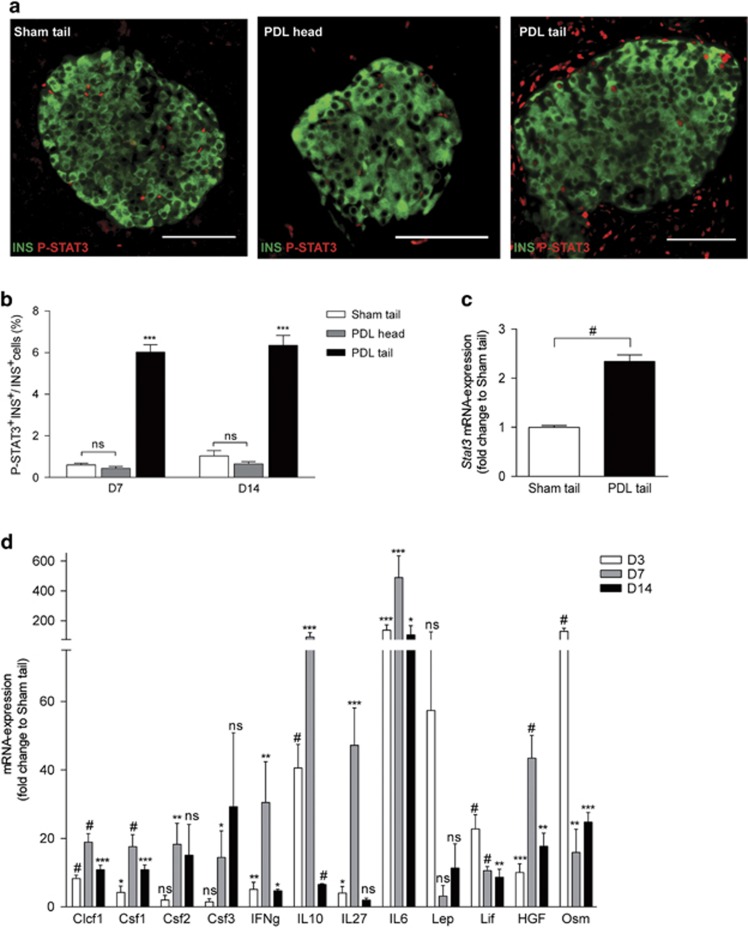
STAT3 expression and activity are stimulated in *β*-cells of PDL pancreas. (**a**) Immunostaining for phospho-STAT3 (Y705) (P-STAT3) and insulin in Sham tail, PDL head and PDL tail. (**b**) Quantification of data in **a**: percentage of P-STAT3^+^ INS^+^ cells at day 7 and 14 (D7 and D14) postsurgery (mean±S.E.M., *n*=3, ***P<0.001, ns: *P>*0.05, two-way ANOVA). (**c**) *Stat3* mRNA in *β*-cells isolated at D7 from the tail of Sham- and PDL pancreas of *MIP-RFP* mice. Data are expressed as fold change *versus* Sham (=1) (*n*=3, ^#^*P<*0.0001, Sham tail *versus* PDL tail by unpaired two-tailed *t*-test). (**d**) STAT3-activating factors whose expression increased 10-fold or more in PDL pancreas. Data represent the fold change in expression of each factor in PDL tail as compared with its expression in Sham tail (1) (D3: *n*=4, D7: *n*=4, D14: *n*=3, *P<0.05, **P<0.005, ***P<0.001, ^#^*P<0.0001*, ns: *P>*0.05, Sham tail *versus* PDL tail by unpaired two-tailed *t*-test)

Eight weeks old BALB/cJRj mice underwent PDL or sham surgery and the expression of Stat3 transcript and protein was evaluated at day (D) 7 and 14 post surgery. STAT3 protein was detected in islets in the tail of ligated pancreas (PDL tail), in the tail of sham pancreas (Sham tail) and in the non-ligated head of PDL pancreas (PDL head) (Supplementary Figure S1a). To study STAT3 activation, the expression of phosphorylated STAT3^Y705^ (from hereon called P-STAT3) was examined using a P-STAT3^Y705^-specific antibody. Significantly more P-STAT3^+^ nuclei were identified among INS^+^ *β*-cells of PDL tail at D7 and D14 following surgery, as compared with PDL head or Sham tail (*n*=3; *P*≤0.005) (Figures 1a and b). P-STAT3^+^ nuclei were also observed among glucagon (GCG)^+^ *α*-cells (Supplementary Figure S1b and Cai *et al.*^34^) and among keratin (KRT)19^+^ duct cells (Supplementary Figure S1c) of PDL tail. Although the abundance of *Stat3* mRNA in total PDL tail pancreas was similar to Sham tail and PDL head pancreas(data not shown), *β*-cells isolated from MIP-mRFP D7 PDL tail pancreas contained twice as much *Stat3* transcript compared with *β*-cells isolated from Sham tail pancreas (Figure 1c), suggesting *β*-cell-specific increase of *Stat3* gene expression.

### PDL stimulates the expression of STAT3-activating cytokines and growth factors

Next, the nature of PDL-induced STAT3 activators was investigated. PDL is characterized by acinar cell loss and formation of neoplastic ducts, accompanied by a local influx of inflammatory CD45^+^ cells.^21^ The cytokine profile of PDL pancreas has been partially characterized.^24, 25^ A literature search revealed 35 potential activators of STAT3 (Table 1) and their mRNA abundance was investigated under our experimental conditions. The expression of 12 of these factors increased more than 10-fold in PDL *versus* sham tail (Figure 1d). Among these, IL6 was most strongly induced (488-fold increase), whereas transcript levels of *Clcf1*, *Csf1*, *Csf2*, *Csf3*, *Ifng*, *IL10*, *IL27*, *Lep*, *Lif*, *Hgf* and *Osm* also increased (Figure 1d). Cytokines with moderately increased transcript level (between 1- and 10-fold) included *Igf1*, *IL2*, *IL7*, *IL11*, *IL15*, *IL19*, *Cntf* and *Pdgfb* (Supplementary Figure S2a). The expression of three factors (*Ctf1*, *Egf* and *Vegfa*) remained either unchanged or decreased in PDL *versus* Sham tail (Supplementary Figure S2b). Finally, a group of nine cytokines known to activate STAT3, including *IL9*, *Ctf2*, *Ifna4*, *Ifna5*, *IL20*, *IL21*, *IL22*, *IL24* and *IL31*, remained undetected. PDL thus creates an inflammatory microenvironment, characterized by increased expression of STAT3-activating cytokines among which IL6 is most prominent.

### Cycling *β*-cells rarely activate STAT3

**Figure 2:**
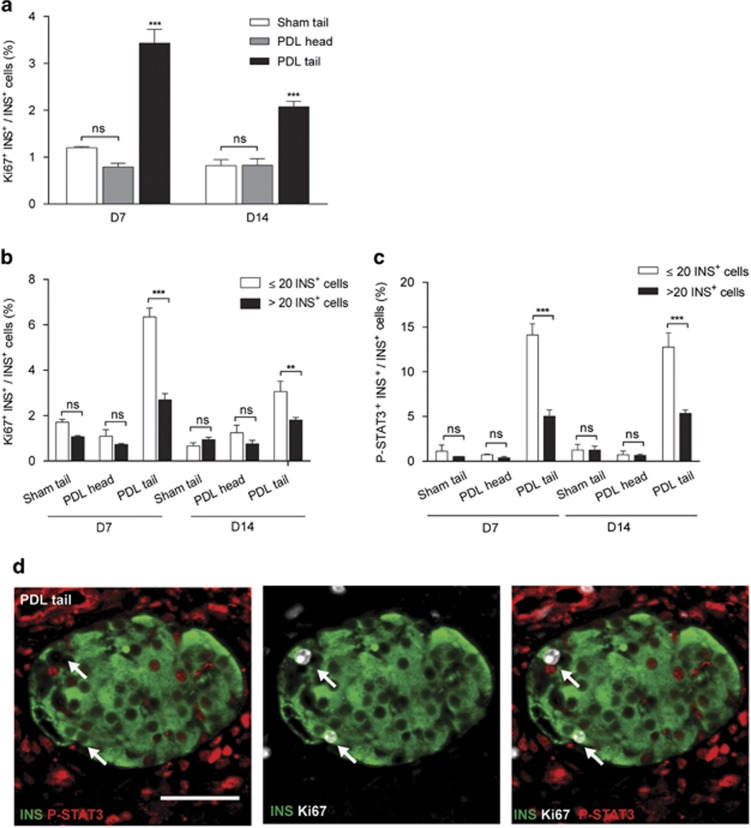
Ki67^+^ *β*-cells differ from P-STAT3^+^ *β*-cells in PDL pancreas. (**a**) *β*-Cells with active cell cycle (Ki67^+^) at D7 and D14 post surgery (mean±S.E.M., D7: *n*=3, D14: *n*=4-6, ***P<0.001, ns: *P>*0.05, PDL tail *versus* Sham tail or PDL head by two-way ANOVA). (**b**) Proliferating *β*-cells (Ki67^+^ INS^+^) and (**c**) *β*-cells with activated STAT3 (P-STAT3^+^ INS^+^) cells in small (≤20 INS^+^ cells) and large (>20 INS^+^ cells) islets, at D7 and D14 post surgery (mean±S.E.M., D7: *n*=3, D14: *n*=4-6, **P<0.005, ***P<0.001, ns: *P>*0.05, small *versus* large islets in PDL and Sham by unpaired two-tailed *t*-test). (**d**) Ki67 and P-STAT3 in *β*-cells in PDL pancreas. Ki67^+^ *β*-cells are P-STAT3^−^ (white arrows)

STAT3 supports cell cycling of B-lymphocytes and several cancer cell types.^35, 36, 37^ Therefore, we examined expression of P-STAT3 in actively cycling Ki67^+^ INS^+^ *β*-cells. At D7 and D14, respectively, 2.8- and 2.5-fold more Ki67^+^ INS^+^ cells were observed in PDL as compared with Sham tail (*n*=3–6, *P*<0.005) (Figure 2a). These data confirm our earlier reports^21, 34, 38^ and indicate that, in PDL tail, both *β*-cell cycle and the number of *β*-cells with activated STAT3 are increased. In line with our previous observations, Ki67^+^ *β*-cells and P-STAT3^+^ *β*-cells were more abundant in small islets of <20 *β*-cells as compared with larger islets at D7 and D14 post PDL (Figure 2b). However, no P-STAT3^+^Ki67^+^ *β*-cells were observed in PDL tail at D7 or D14 (Figure 2d and Table 2). The close to zero probability to find a P-STAT3^+^Ki67^+^ *β*-cell in PDL tail, as calculated by the Wilson score confidence interval (95%) (*P*<0.001, Table 2), indicates that the population of cycling *β*-cells does not overlap with the population of *β*-cells with activated STAT3. This phenomenon appears specific for *β*-cells, as P-STAT3^+^ Ki67^+^ duct- and *α*-cells were observed under the same experimental conditions (Supplementary Figure S2c).

### Conditional *Stat3* knockout increases *β*-cell cycling in PDL pancreas

**Figure 3:**
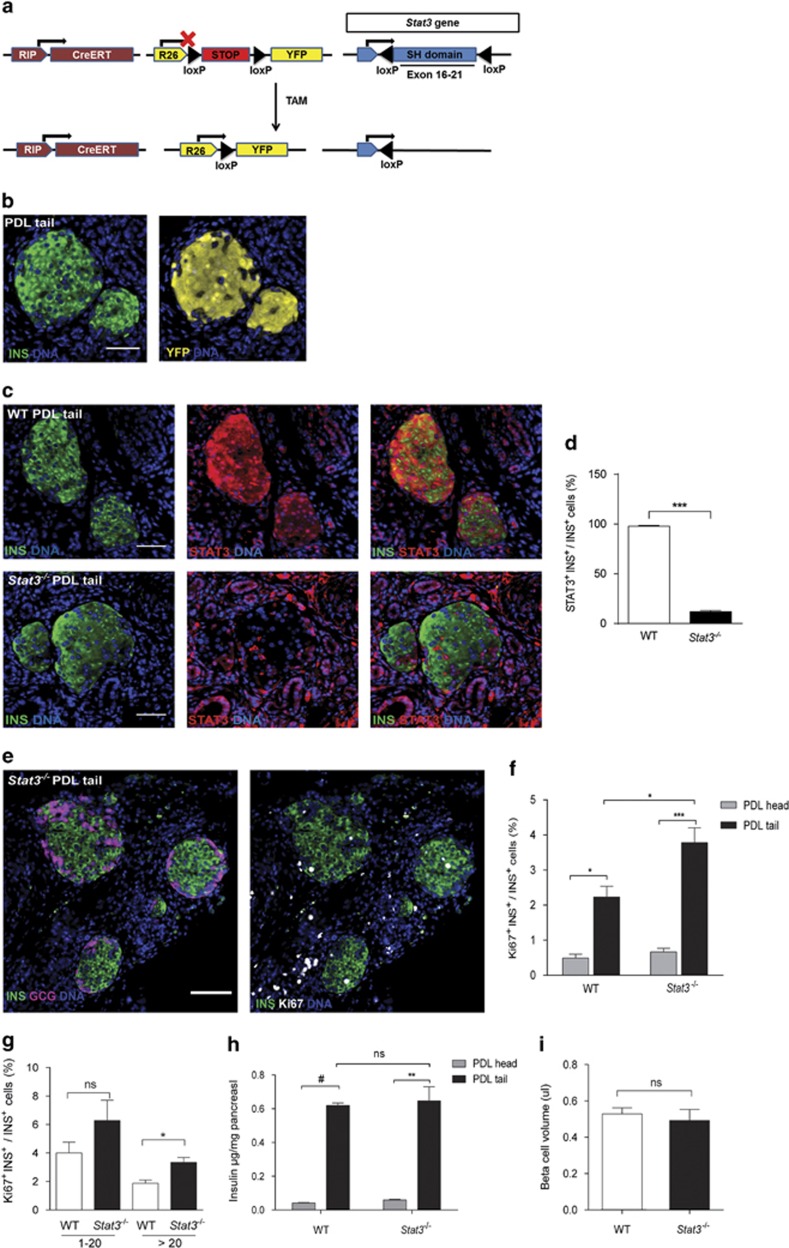
*β*-Cell-specific *Stat3* knockout stimulates *β*-cell cycling after PDL. (**a**) Schematic illustration of the inducible transgenic mouse model, RIP (rat insulin gene promotor), CreERT (causes recombination fused to TAM-inducible estrogen receptor), R26 (Rosa26 promotor), loxP (locus of crossing over P1), STOP (transcriptional STOP sequence), YFP, TAM. (**b**) Recombination efficiency as shown by immunostaining for YFP in INS+ cells in PDL tail of RipCre^ERT^R26^YFP^ mouse. (**c**) Immunostaining for STAT3 in PDL tail from WT *versus Stat3*^*−/−*^ mice, quantified in (**d**) as the percentage of STAT3^+^ INS^+^ cells in PDL tail from WT and *Stat3*^*−/−*^ mice. **(e)** Cycling *β*-cells (Ki67^+^ INS^+^) were observed in the islets of *Stat3*^*−/−*^ mice that appeared normal based on the distribution of *β*- and *α*-cells (GCG^+^). **(f)** *β*-Cell cycling (Ki67^+^ INS^+^) in PDL head and tail pancreas from WT and *Stat3*^*−/−*^ mice at D14 post surgery. (**g**) Percentage INS^+^ cells that express Ki67^+^, in small (≤20 INS^+^ cells) and large (>20 INS^+^ cells) islets of PDL head and tail pancreas from WT and *Stat3*^*−/−*^ mice at D14 post surgery. (**h**) Total insulin content (*μ*g insulin per mg tissue) in PDL head and tail pancreas from WT and *Stat3*^*−/−*^ mice. (**i**) *β*-Cell volume (*μ*l) in PDL tail pancreas from WT and *Stat3*^*−/−*^ mice. **(d** and **f**–**i)** mean±S.E.M., in (**d** and **f**–**h**) *n*=4, in (**i**) *n*=3–4, *P<0.05, **P<0.005, ***P<0.001, ^#^*P*<0.0001, ns: *P>*0.05, WT *versus Stat3*^*−/−*^ by unpaired two-tailed *t*-test

The functional role of STAT3 in *β*-cells of PDL pancreas was investigated in *RIP*^*CreERT*^*;R26*^*YFP*^*;Stat3*^*lox/lox*^ mice (Figure 3a) that received tamoxifen (TAM) at 5 weeks of age, followed by a 14 days washout period. PDL was performed at 8 weeks of age and analysis was carried out 2 weeks later. As the efficiency of recombination in *β*-cells was high (89.9±1.11% of INS^+^ cells were YFP^+^) (Figure 3b), *RIP*^*CreERT*^*;R26*^*YFP*^*;Stat3*^*lox/lox*^ mice that received TAM are hereafter referred to as *Stat3*^*−/−*^. Accordingly, an 85.8% reduction in STAT3^+^ *β*-cells was observed in PDL tail of *Stat3*^*−/−*^ mice *versus* wild-type (WT) littermates (*n*=4) (Figures 3c and d). Bodyweight, glycemia and glucose tolerance (as determined by IPGTT) were similar in *Stat3*^*−/−*^ and WT, both before and after PDL (Supplementary Figures S3a–c). The islet architecture in *Stat3*^*−/−*^ mice appeared normal (Figure 3e). In addition, the percentage of Ki67^+^ *β*-cells was similar in PDL heads of WT and *Stat3*^*−/−*^ mice (Figure 3f). The percentage of Ki67^+^ *β*-cells in PDL tail as compared with PDL head was 4.5-fold higher In WT mice, (*n*=4, *P*<0.05) and 5.7-fold in *Stat3*^*−/−*^ mice (*n*=4, *P*<0.005) (Figure 3f), indicating that STAT3 in *β*-cells is dispensable for *β*-cell cycling after PDL. Notably, PDL-induced *β*-cell cycle was amplified by 70% in the absence of STAT3 (Figure 3f). As Ki67^+^ *β*-cells are most prominent in small islets of PDL tail in WT mice^38, 39^ (Figure 2c), increased *β*-cell cycling observed in *Stat3*^*−/−*^ pancreas could conceivably be caused by an increased amount of small islets. However, the distribution of small, medium and large islets in *Stat3*^*−/−*^ mice was similar to that in the WT mice (Supplementary Figure S4a). *β*-Cell cycling was only significantly increased in large islets in *Stat3*^*−/−*^ compared with WT mice. (Figure 3g). Despite this increase, insulin content and *β*-cell volume was similar in PDL tail of *Stat3*^*−/−*^ and WT mice (Figures 3h and i). The percentage of Ki67^+^ *α*-cells nor the *α*- to *β*-cell ratio were altered in PDL tail of *Stat3*^*−/−*^ compared with WT mice (Supplementary Figures S4b and c).

### STAT3 activation by rIL6 lowers *β*-cell cycling in PDL pancreas

**Figure 4:**
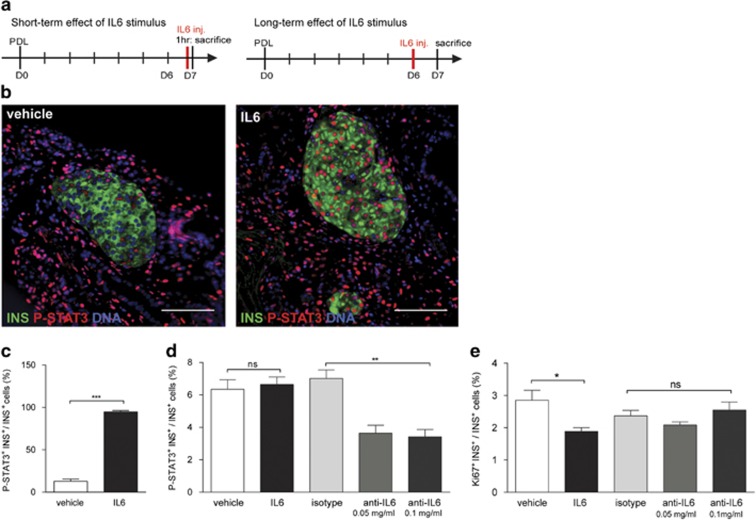
Recombinant IL6 stimulates STAT3 activation and blunts *β*-cell cycling in PDL pancreas. (**a**) Left: short-term effect of recombinant (r) IL6, injected at D7 post surgery into PDL pancreas that was collected 1 h post injection. Right: long-term effect of recombinant (r) IL6 injected at D6 post surgery into PDL pancreas that was collected 24 h post injection. (**b**) Immunostaining for P-STAT3 and insulin (INS) in PDL tail pancreas 1 h after (r)IL6 or vehicle injection at D7 post PDL. (**c**) Percentage of P-STAT3^+^ *β*-cells in PDL tail 1 h after vehicle or (r)IL6 injection (mean±S.E.M., *n*=4, ***P<0.001, vehicle *versus* (r)IL6-injected PDL tail pancreas, by unpaired two-tailed *t*-test). (**d**) Percentage P-STAT3^+^ *β*-cells 24 h after vehicle, (r)IL6, isotype or anti-IL6 injection in PDL tail at D7 post PDL (*n*=4, **P<0.005, ns: *P>*0.05, vehicle *versus* (r)IL6-injected PDL tail pancreas and isotype *versus* anti-IL6-injected PDL pancreas, two-way ANOVA). (**e**) Cycling *β*-cells (Ki67^+^ INS^+^) in PDL tail injected with vehicle, (r)IL6, isotope or anti-IL6 at D7 post surgery (mean±S.E.M., *n*=4, *P<0.05, ns: *P>*0.05, vehicle *versus* (r)IL6-injected PDL tail pancreas and isotype *versus* anti-IL6-injected PDL pancreas, two-way ANOVA)

As IL6 is a potent activator of STAT3,^40^ recombinant mouse IL6 (rIL6) was injected in the tail of D7 PDL pancreas, in order to evaluate the effect of increased STAT3 activation on *β*-cell proliferation. As STAT3 protein has a short half-life,^41^ pancreas was collected 1 h after injection (Figure 4a). *β*-Cells (95±3%) contained P-STAT3 in rIL6-injected PDL tail as compared with only 13±5% following vehicle injection (Figures 4b and c). Twenty-four hours after rIL6 injection, the percentage of P-STAT3^+^ *β*-cells in PDL tail had returned to baseline (Figure 4d). However, at this time point, the percentage of Ki67^+^ *β*-cells in rIL6-injected PDL tail was significantly lower when compared with vehicle control (Figure 4e). These data suggest that activation of STAT3 by exogenous IL6 blunts PDL-induced *β*-cell cycling. To determine whether endogenous IL6 contributed to STAT3 activation in PDL tail, an IL6-neutralizing antibody was injected in PDL tail at D6 post surgery. Twenty-four hours after injection, the percentage of P-STAT3^+^ *β*-cells in PDL tail was 50% lower in anti-IL6-injected as compared with isotype-injected pancreas (Figure 4d), indicating that endogenous IL6 contributes to STAT3 activation in *β*-cells. However, *β*-cell cycling was not significantly increased in anti-IL6-injected PDL tail compared with isotype-injected PDL tail (Figure 4e), suggesting that a 50% reduction in STAT3 activation in *β*-cells is not sufficient to activate the *β*-cell cycle as compared with deletion of a functional STAT3 in 90% of *β*-cells. Alternatively, a STAT3-mediated delay to initiate *β*-cell cycling may have preceded injection of anti-IL6 antibody.

### STAT3 protects *β*-cells from DNA damage in PDL

**Figure 5:**
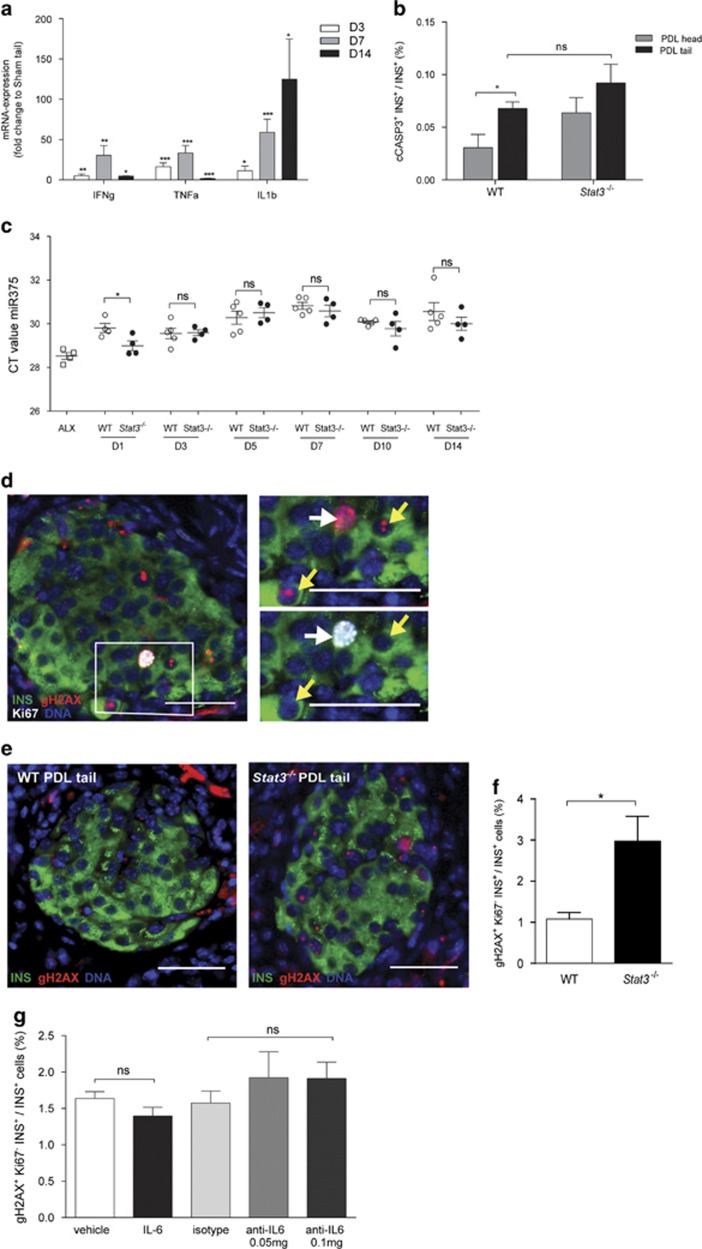
STAT3 protects *β*-cells from DNA damage in PDL pancreas. (**a**) Abundance of mRNA encoding inflammatory cytokines (*Ifng*, *Tnfa* and *Il1b*) in PDL tail. Data are expressed as fold change compared with transcript level in Sham tail (1) (D3: *n*=4, D7: *n*=4, D14: *n*=3, *P<0.05, **P<0.005, ***P<0.001, ^#^*P<*0.0001, ns: *P>*0.05, Sham tail *versus* PDL tail by unpaired two-tailed *t*-test). (**b**) Percentage *β*-cell apoptosis (cCASP3^+^ INS^+^) in PDL head and tail pancreas from WT and *Stat3*^*−/−*^ mice at D14 post surgery (*n*=4, *P<0.05, ns: *P>*0.05, WT *versus Stat3*^*−/−*^, two way ANOVA). (**c**) Abundance of miR375 in plasma from WT and *Stat3*^*−/−*^ mice at D1, D3, D5, D7, D10 and D14 post PDL surgery and in plasma of positive control for *β*-cell death 24 h after Alloxan injection (ALX). Data are expressed as Ct values. WT, *n*=5; *Stat3*^*−/−*^, *n=4*; *P<0.05, ns: *P>*0.05, WT *versus Stat3*^*−/−*^ by unpaired two-tailed *t*-test) (**d**) Immunostaining for Ki67 and gH2AX in PDL tail pancreas from *Stat3*^*−/−*^ mice showing two forms of gH2AX nuclear staining: homogenous nuclear labeling in proliferating *β*-cells (white arrows) and nuclear foci (yellow arrows). (**e**) gH2AX in PDL tail from WT and *Stat3*^*−/−*^ mice at D14 post surgery. (**f**) Percentage non-cycling *β*-cells with DNA damage (gH2AX^+^ Ki67^−^ INS^+^) in PDL tail from WT and *Stat3*^*−/−*^ mice at D14 post surgery (*n*=4, *P<0.05, ns: *P>*0.05, WT *versus Stat3*^*−/−*^, by unpaired two-tailed *t*-test). (**g**) Percentage non-cycling *β*-cells with DNA damage (gH2AX^+^ Ki67^−^ INS^+^) in PDL tail pancreas injected with vehicle, (r)IL6, isotype or anti-IL6 at D7 post surgery (*n*=4, *P<0.05, ns: *P>*0.05, two-way ANOVA)

As the increased number of cycling *β*-cells observed in *β*-cell-specific *Stat3*^*−/−*^ mice was not accompanied by an increase in pancreatic insulin content and *β*-cell volume (Figures 3h and i), we hypothesized that *β*-cells may be experiencing stress under these conditions. Indeed, the mRNA expression of deleterious cytokines *Il1b*, *Ifng* and *Tnfa*^42^ were significantly higher in PDL tail at D3, 7 and 14 following surgery, with the most prominent induction at D7 (Figure 5a). Although *β*-cells that expressed the apoptosis marker cleaved caspase 3 could be identified in WT and *Stat3*^*−/−*^ PDL tail, their percentage was very low (<0.1% of INS+ cells) (Figure 5b). In PDL tail of *Stat3*^*−/−*^ mice, the increase in percentage of cleaved caspase 3^+^ *β*-cells, as compared with WT, was not statistically significant (Figure 5b). However, as late-apoptotic cells are likely to be cleared quickly, we measured levels of the *β*-cell death marker miR375 via quantitative reverse transcription PCR in plasma of WT and *Stat3*^*−/−*^ mice at D1–D14 post PDL surgery. MiR375 is a *β*-cell-specific microRNA and is discharged in the circulation at levels that are proportionate to the number of necrotic or apoptotic *β*-cells.^43, 44^ Although miR375 could be detected at all time points following PDL, its level at D1 was highest in *Stat3*^*−/−*^ compared with WT mice. These data suggest elevated *β*-cell loss in PDL pancreas of *Stat3*^*−/−*^ mice (Figure 5c). To assess DNA damage in *β*-cells in PDL pancreas of *Stat3*^*−/−*^ and WT mice, expression of histone *γ*-H2AX (gH2AX) was investigated. Phosphorylation of gH2AX at sites of DNA double-strand breaks aids recruitment of DNA repair factors^45^ and is typified by focal nuclear staining for gH2AX. In contrast, homogenous or pan-nuclear staining for gH2ax is observed in nuclei of cells with single-strand breaks induced by UV irradiation, during nucleotide excision repair, in DNA fragmentation during apoptosis^46^ but also in nuclei of replicating cells with undamaged DNA.^47, 48, 49^ In WT and *Stat3*^*−/−*^ PDL, immunostaining for gH2AX revealed *β*-cells whose nuclei either displayed 1 or 2 positive nuclear foci (yellow arrowheads in Figure 5d) and Ki67+ *β*-cells whose nuclei displayed homogenous positive nuclear gH2AX staining (white arrow in Figure 5d). Homogenous staining for gH2AX was found in approximately half of Ki67^+^ *β*-cells in PDL tail (51% in *Stat3*^*−/−*^ and 47% in WT mice) (Figure 5f). When these cells were excluded from our DNA damage analysis, *γ*-H2AX^+^ Ki67^−^ *β*-cells were 2.7-fold more abundant in PDL tail of *Stat3*^*−/−*^ *versus* WT mice (Figures 5e and f). The high efficiency of *Stat3* deletion (90%) in *Stat3*^*−/−*^ mice appeared crucial for the effect on DNA damage, as 50% inhibition of STAT3 activity by injection of anti-IL6 antibody into PDL pancreas did not affect the percentage of gH2AX^+^ Ki67^−^ *β*-cells (Figure 5g). Conversely, injection of IL6 in order to increase STAT3 activation and diminish DNA damage in PDL pancreas did not significantly affect the percentage of gH2AX^+^ Ki67^−^ *β*-cells compared with vehicle-injected PDL. These findings suggest that the protective effect of STAT3 on *β*-cell DNA integrity may not only depend on IL6-induced activation of STAT3.

## Discussion

STAT3 signaling is dispensable for normal pancreas development but is involved in malignant processes such as acinar-to-ductal metaplasia^50^ and pancreatic ductal adenocarcinoma.^36, 51^ PDL induces severe injury resulting in massive loss of exocrine acinar cells and acinar-to-ductal metaplasia, accompanied by local inflammation with infiltration of CD45^+^ cells and expression of various cytokines and growth factors.^21, 23, 24, 25^ We previously demonstrated that PDL causes *β*-cell mass expansion, in part attributed to proliferation of both pre-existing and newly formed *β*-cells.^21, 38^ In the present study, we investigated STAT3 activation in PDL and its role in increased *β*-cell proliferation.

Activation of STAT3 is induced by factors specific to the PDL environment.^21, 23, 24, 25^ We identified 20 STAT3-activating factors that are moderately or highly upregulated in ligated pancreas, and that promote *Stat3* gene expression and protein activation specifically in *β*-cells located in small islets. This preferential localization suggests involvement of STAT3 signaling in *β*-cell proliferation, as the latter is predominantly observed within small islets. In addition, transduction of cultured rat *β*-cells with a constitutively active form of STAT3 has been shown to induce a 2.8-fold higher BrdU incorporation rate.^52^ Nevertheless, P-STAT3 and Ki67 did not co-localize in *β*-cells of PDL pancreas, irrespective of islet size, whereas co-localization of Ki67 and P-STAT3 was observed in *α*- and duct cells, suggesting a direct role for STAT3 in the cell cycle of these latter cell types only.

To address STAT3 function in *β*-cells, we generated mice that allow efficient, inducible and *β*-cell-specific *Stat3* knockout. These mice displayed normal bodyweight and glycemic control, irrespective of PDL. This is in contrast to *RIP*^*Cre*^-induced deletion of *Stat3* in developing *β*-cells and in insulin-expressing hypothalamic neurons – where it disrupts leptin signaling – which results in appetite gain and mild obesity.^15, 16^ Together with the fact that *Cre* expression *per se* disturbs *β*-cell function,^53^ these off-target effects explain why *RIP*^*Cre*^*;Stat3*^*lox/lox*^ mice become obese and glucose intolerant.^15, 16^ Our data suggest that, within the time frame of our study, that is, 4–5 weeks after recombination, deletion of *Stat3* from adult *β*-cells or hypothalamic neurons (as shown by yellow fluorescent protein (YFP) expression by hypothalamic neurons; Supplementary Figure S5) does not interfere with normal physiology and glucose handling. We therefore assume that the effects of STAT3 inactivation in our model are *β*-cell specific rather than systemic.

As *Stat3*-depleted *β*-cells still express Ki67 in PDL pancreas, STAT3 signaling seems not to be required for *β*-cell cycling. On the contrary, *Stat3* deletion from *β*-cells increased the percentage of Ki67^+^ *β*-cells in PDL pancreas. Consequently, STAT3 appears to inhibit or at least delay the initiation of the *β*-cell cycle in injured pancreas. In-depth analysis of STAT3 activation dynamics and activation of the *β*-cell cycle is required to define this inhibition or delay and its effects. A similar role for STAT3 in growth arrest has been identified in monocytes.^40^ In addition, in T-lymphocytes, STAT3 inhibits the proliferative response to IL-2,^54^ whereas IL6-mediated activation of STAT3 inhibits hepatocyte proliferation^55^ and growth of carcinoma and myeloid leukemia cells.^40^ As the percentage of Ki67^+^ *β*-cells did not increase in PDL head of pancreas in *Stat3*^*−/−*^ mice, cytokines or growth factors produced locally in PDL tail may signal via STAT3 and prevent excessive *β*-cell proliferation specifically in PDL tail. As a higher number of *β*-cells in small islets, a hotspot for *β*-cell proliferation after PDL, displayed STAT3 activation, we assume that STAT3 limits or delays excessive *β*-cell cycling in pro-proliferative environments. *Stat3* deletion allowed increased *β*-cell proliferation, mainly in large islets. Possibly, activation of *β*-cell division in *Stat3*^*−/−*^ mice was less pronounced in small islets, because this location already is a privileged site for *β*-cell division. It is also a site where new STAT3-competent *β*-cells are formed by differentiation, thus escaping *Stat3* deletion that was induced in pre-existing *β*-cells before surgery.^38^

Increased *β*-cell cycling without an associated increase in insulin content and *β*-cell volume in PDL pancreas of *Stat3*^*−/−*^ mice suggests that not all actively cycling *β*-cells complete their cell cycle. However, the percentage Ki67^+^ *β*-cells that contain the G2-M marker phosphohistone H3 was 30% in both *Stat3*^*−/−*^ and WT mice (data not shown). By consequence, increased *β*-cell death in PDL pancreas of *Stat3*^*−/−*^ mice is an alternative explanation for increased *β*-cell cycling without a concomitant increase in *β*-cell volume. An increase in *β*-cell death was demonstrated by analysis of plasma miR-375 levels early after PDL in *Stat3*^*−/−*^ mice.

Exposure to the pro-inflammatory cytokines IL1b and IFNg that both are present in PDL pancreas, causes nitric oxide-mediated DNA damage in *β*-cells that contain reduced amounts of insulin and display suppressed glucose-stimulated insulin secretion.^56, 57^ The cytokine-induced DNA damage that is characterized by an increased number of *β*-cells with phosphorylated gH2AX leads to *β*-cell death.^58^ The percentage of *β*-cells expressing gH2AX was significantly increased in mice with *β*-cell-specific *Stat3*^*−/−*^. Therefore, STAT3 may serve as (part of) a mechanism to secure DNA integrity before cell cycling. In regenerating liver, STAT3 lowers hepatocyte cycling by increased expression of p21^Cip1^,^55^ coinciding with the upregulation of several DNA repair enzymes.^59^ Mice that undergo PDL harbor increased levels of inflammatory cytokines in the PDL tail, while displaying normal glycemic control and only low numbers of apoptotic (cleaved caspase3^+^) *β*-cells, indicative for a very tight regulation and protection of *β*-cells from these deleterious factors. Protection from cytokine-induced *β*-cell apoptosis has been described for CNTF and humanin, signaling through STAT3.^60, 61^ We hypothesize the existence of a similar protective role for STAT3 against inflammatory cytokines in PDL.

To further support our observations in *Stat3* knockout mice, we injected recombinant IL6 into the ligated part of the pancreas, thereby efficiently boosting local STAT3 signaling in *β*-cells, as acinar atrophy and shrinkage of the tissue allows for easy diffusion of the cytokines. Increased activation of STAT3 was sufficient to blunt PDL-induced activation of *β*-cell cycling, similar to regenerating liver.^55^ DNA damage, however, did not decrease on IL6 injection, likely because basal DNA damage in *β*-cells is as low as 1.6% in G0 *β*-cells of PDL pancreas. In addition, IL6-activated STAT3 can be negatively regulated by SOCS3 that inhibits JAK activation by binding to gp130 through its SH2 domain. *β*-Cell-specific *Socs3* knockout maintains STAT3 activation and, consequently, the expression of *Bcl-xL* that reduces streptozotocin-induced toxicity and apoptosis.^62^ In PDL pancreas, STAT3 activation may be short term and its signaling may be inhibited through negative feedback by SOCS3.^60^ The abundance of STAT3 appears crucial, as a 50% reduction by anti-IL6 injection was insufficient to increase *β*-cell cycling or DNA damage in PDL pancreas, as compared with the 90% reduction in *Stat3*^*−/−*^ mice. Factors other than IL6 may thus contribute to the control of STAT3 activity in *β*-cells in ligated pancreas. Other potential STAT3-activating factors that are increased in PDL pancreas and for which receptors are expressed by *β*-cells include CNTF,^60^ Leptin,^63^ IL10,^64^ HGF,^65^ IGF^66^ and PDGF.^67^ Although these factors potentially modulate STAT3 in *β*-cells in PDL pancreas, still many other cytokines and signaling pathways may participate in the regulation of *β*-cell proliferation and survival in PDL. Therefore, PDL offers an interesting tool for the identification of signaling pathways and factors that influence *β*-cell survival and growth in an inflammatory environment.

In conclusion, our data indicate that STAT3 protects *β*-cells from excessive cell cycling and from deleterious effects exerted by cytokines that are inherent to the inflammatory environment of duct-ligated pancreas.

## Materials and Methods

### Animals

All experiments were performed in accordance with the guidelines of the ethical committee of the Vrije Universiteit Brussel. Mice were housed in micro-isolator cages, maintained on a 12/12 h light–dark cycle and fed a standard rodent diet.

### Mouse manipulation

PDL was performed as described^21, 68^ on 8- to 9-week-old male mice. For IL6 and anti-IL6 intrapancreas injection, 8-week-old BALB/cJRj male mice (Janvier, Saint Berthevin, France) were anesthetized by i.p. injection of 3.5 mg ketamine+0.5 mg xylazine per kg bodyweight. The ligated pancreas was exposed through a lateral incision and injected with either 30 μl of 0.1 mg/ml carrier-free rIL6 (R&D Systems, Minneapolis, MN, USA), vehicle (0.9% NaCl), 0.1 or 0.05 mg/ml anti-IL6 (clone MP520F3, Hycult Biotech, Uden, The Netherlands) or rat IgG (Vector Laboratories, Burlingame, CA, USA). TAM (Sigma-Aldrich, St. Louis, MO, USA) was prepared and administered as described.^38^

### *β*-Cell-specific Stat3 knockout mice

*β*-Cell-specific *Stat3* knockout mice were generated by crossing *RIP*^*CreER*^*;R26*^*YFP*^ mice with *Stat3*^*lox/lox*^ mice. *RIP*^*CreER*^*;R26*^*YFP*^ mice express a fusion protein of Cre recombinase and a mutated TAM responsive estrogen receptor under control of the rat insulin promoter (RIP) in addition to conditional YFP reporter expression under control of the *Rosa26* promotor (R26).^69^ In *Stat3*^*lox/lox*^ mice, exon 16–21 encoding the SH-domain of the STAT3 protein is flanked by loxP sites. As control mice, CreER-deficient *Stat3*^*lox/lox*^ littermates were used. Genotyping was performed on tail snips using primers for *Cre* (forward 5′-CCTGTTTTGCACGTTCACCG-3′, reverse 5′-ATGCTTCTGTCCGTTTGCCG-3′), *YFP* (forward 5′-AAAGTCGCTCTGAGTTGTTA-3′, reverse 5′-GGAGCGGGAGAAATGGATATG-3′), splice acceptor (5′-CATCAAGGAAACCCTGGACTACT-3′) and for the *Stat3* floxed allele (forward 5′-ATTGGAACCTGGGACCAAGTG-3′, reverse 5′-ACATGTACTTACAGGGTGTGT-3′). *RIPCre*^*ERT*^*;R26*^*YFP*^*; Stat3*^*lox/lox*^ mice or WT *RIPCre*^*ERT*^ negative littermates received 4 mg TAM, repeated once every other day, for five injections in total, followed by a 14-day washout period and PDL surgery. PDL head and PDL tail was collected 14 days post surgery.

### Metabolic follow-up

Mice were fasted for 2 h before measurement of glycemia. For IPGTT, mice were fasted for 16 h before i.p. injection with glucose (2 g per kg body weight). Blood glucose concentration was measured from tail vein blood with a portable glucometer (Glucomen Lx Plus, Menarini, Florence, Italy). Total pancreas insulin content was determined using a mouse insulin radioimmunoassay kit (Linco Research, St. Charles, MO, USA).

### RNA analysis

PDL and Sham tissues were dissociated to single cells using collagenase (0.8 mg/ml, Sigma-Aldrich) and trypsin (1 mg/ml, Sigma-Aldrich), passed through a 66-μm filter and resuspended in isolation medium (Lonza, Basel, Switzerland). *β*-Cells were isolated by sorting on a FACSDiva 6.0 (Becton Dickinson, Franklin Lakes, NJ, USA) RFP^+^ cells from PDL'ed *MIP*^*mRFP*^ mice followed by a second round of sorting using Zn^2+^ chelator 6-methoxy-8-p-toluenesulfonamido-quinolone (Thermo Fisher Scientific, Waltham, MA, USA) as *β*-cell probe. Total RNA was isolated from cells (RNeasy, Qiagen, Venlo, The Netherlands) or tissue (TRIzol, Thermo Fisher). RNA analysis of cells or tissue was as described,^38^ using primers listed in Supplementary Table S1.

### Protein analysis

Samples for immunostaining were fixed overnight at 4 °C in 10% neutral buffered formalin, embedded in paraffin and sectioned at 5 μm. Primary antibodies used were guinea pig polyclonal anti-insulin and rabbit polyclonal anti-glucagon (Diabetes Research Center, Vrije Universiteit Brussel, Brussels, Belgium), rabbit polyclonal anti-Ki67 (eBioscience, San Diego, CA, USA), rat polyclonal anti KRT19 (Developmental Studies Hybridoma Bank, University of Iowa, Iowa City, IA, USA), rabbit monoclonal anti-phosphoSTAT3 (P-STAT3) (phospho Y705) and anti-*γ* H2AX (gH2AX) (phospho S139), goat polyclonal anti-GFP, rabbit polyclonal anti-cleaved caspase3 (Asp175) (all from Abcam, Cambridge, UK) and mouse monoclonal anti-STAT3 (Cell Signaling Technology, Danvers, MA, USA), Staining for GFP, KRT19, glucagon, P-STAT3, STAT3 and gH2AX required heat-mediated antigen retrieval with 10 mM citrate pH 6.0. Cyanine- and AlexaFluor-labeled secondary antibodies were from Jackson ImmunoResearch, Newmarket, UK. Nuclei were stained with Hoechst 33342. Images were acquired with a BX-UCB fluorescence microscope (Olympus, Tokyo, Japan) equipped with an Orca r^2^ camera (Hamamatsu Photonics, Hamamatsu, Japan). Images were analyzed with SmartCapture 3 (Digital Scientific UK, Cambridge, UK). *β*-Cell proliferation, STAT3 activation, *β*-cell apoptosis and DNA damage in *β*-cells were analyzed by determining the proportion of insulin-positive cells co-expressing Ki67, phospho-STAT3, cleaved caspase-3 or gH2AX, respectively. At least 2000 *β*-cells were quantified per sample. Quantification was done by manual counting using the cell counter plugin and SmartCaptureLite plugin (Digital Scientific UK) in ImageJ (NIH, Bethesda, MA, USA). The total *β*-cell volume was analyzed as previously described.^21, 38, 70^ Images were captured with an inverted microscope (Nikon Instruments, Amstelveen, The Netherlands) equipped with a Hamamatsu digital camera c10600 (Olympus) or with a multiphoton microscope (LSM710 NLO with a TiSa laser, Zeiss, Thornwood, NY, USA) and analysed using Smartcapture 3 (version 3.0.8) NIS AR2.30 Imaging Software (Nikon France, Champigny-sur-Marne, France) or Improvision Volocity LE (version 5.0) (PerkinElmer, Waltham, MA, USA) and ImageJ software (https://imagej.nih.gov/ij/).^71^

### miR375 analysis in plasma

Plasma was obtained from tail vein blood from adult WT and *Stat3*^*−/−*^ mice at D1, D3, D5, D7, D10 and D14 post PDL surgery. Analysis of miR375 was performed as described by Roels *et al.*^72^ (In preparation).

### Statistical analysis

Data were analyzed using GraphPad Prism 5 (GraphPad Software, San Diego, CA, USA) and are expressed as mean±S.E.M. of at least three independent experiments. Data were analyzed by unpaired two-tailed Student's *t*-test or with one-way or two-way ANOVA with Bonferroni *post-hoc* test and were considered statistically significant when *P*<0.05. To determine the confidence interval for the proportion of P-STAT3^+^ Ki67^+^ *β*-cells, the Wilson score confidence interval was used. Wilson scores have coverage probabilities close to the nominal confidence level, also for small samples sizes or extreme frequencies close to zero.

## Figures and Tables

**Table 1:** Potential STAT3-activating cytokines and growth factors

**Gene**	**Assay ID**
*IL-6 family*	
* IL-6*	Mm.PT.53.10005566
* IL-11*	Mm.PT.53.9688576
* IL-31*	Mm.PT.53.30234305
* Osm*	Mm.PT.53.10391002
* LIF*	Mm.PT.5328778894
* CNTF*	Mm.PT.5332700675.g
* Ctf1*	Mm.PT.53.5674019
* Ctf2*	Mm.PT.53.10088537
* Clcf1*	Mm.PT.53.10279842
	
*IL-10 family*	
* IL-10*	Mm.PT.53.13531087
* IL-19*	Mm.PT.53.10424485
* IL-20*	Mm.PT.53.30487665
* IL-22*	Mm.PT.53.33202172
* IL-24*	Mm.PT.53.11689874
	
*Interferons*	
* IFNα4*	Mm.PT.53.31028456.g
* IFNα5*	Mm.PT.53.32377263.g
* IFNβ1*	Mm.PT.53.29124254.g
* IFNγ*	Mm.PT.53.13380517
	
*Common* γ*-chain family*	
* IL-2*	Mm.PT.53.11478202
* IL-7*	Mm.PT.53.10325839
* IL-9*	Mm.PT.53.17016816
* IL-15*	Mm.PT.53.33007558.g
* IL-21*	Mm.PT.53.7853071
	
*Growth Factors and hormones*	
* Csf-1*	Mm.PT.53a.32521769
* Csf-2*	Mm.PT.53a.30204640
* Csf-3*	Mm.PT.53.14133107.g
* EGF*	Mm.PT.53.6246680
* HGF*	Mm.PT.53.9088506
* IGF-1*	Mm.PT.53.32726889
* VEGFα*	Mm.PT.53.31754187.g
* PDGFβ*	Mm.PT.53a.32585335
* Lep*	Mm.PT.53.13515402
	
*IL-12 family*	
*IL-27*	Mm.PT.53.12354802
	
*Inflammatory cytokines*	
*IL-1β*	Mm.PT.53a.16901608
Tumor Necrosis Factor *α* (*TNFα*)	Mm.PT.56a.12575861

**Table 2:** P-STAT3^+^ proliferating *β*-cells in PDL and Sham pancreas

	**P-STAT3+ proliferating β**-**cells in PDL and Sham pancreas at 7 and 14 days post surgery**											
	**INS+**		**KI67+INS+**		**P-STAT+INS+**		**P-STAT3+KI67+INS+ [CI]**		**%KI67+**		**%P-STAT3+**	
	**D7**	**D14**	**D7**	**D14**	**D7**	**D14**	**D7**	**D14**	**D7**	**D14**	**D7**	**D14**
PDL tail	3786	4843	103	123	230	304	(0, 0.00051)	(0, 0.00040)	2.72	2.54	6.08	6.28
PDL head	3130	3410	16	21	14	22	(0, 0.00061)	(0, 0.00056)	0.51	0.62	0.45	0.65
SHAM tail	3167	4928	55	48	19	50	(0, 0.00061)	(0, 0.00039)	1.74	0.97	0.6	1.01

Quantification of Ki67+ P-STAT3+ double-positive *β*-cells in PDL tail and head, and Sham tail at 7 and 14 days post surgery. INS^+^, number of *β*-cells quantified; Ki67^+^INS^+^, number of proliferating *β*-cells quantified; P-STAT3^+^INS^+^, number of P-STAT3-positive *β*-cells; P-STAT3^+^Ki67^+^INS^+^ [CI], number of P-STAT3-positive and Ki67-positive (proliferating) *β*-cells and the computed Wilson confidence interval, *n*=3

## Abbreviations

Stat3: signal transducer and activator of transcription 3
PDL: partial pancreatic duct ligation
RT-PCR: reverse transcription PCR
R26: Rosa 26 locus
RIP: rat insulin promoter
TAM: tamoxifen
YFP: yellow fluorescent protein
