# Supplementary figures and images for: STAT3 modulates β-cell cycling in injured mouse pancreas and protects against DNA damage
Source: Cell Death Dis. 2016 Jun 23;7(6):e2272–. doi: 10.1038/cddis.2016.171 (PMC5143397; doi:10.1038/cddis.2016.171)

Figure S1

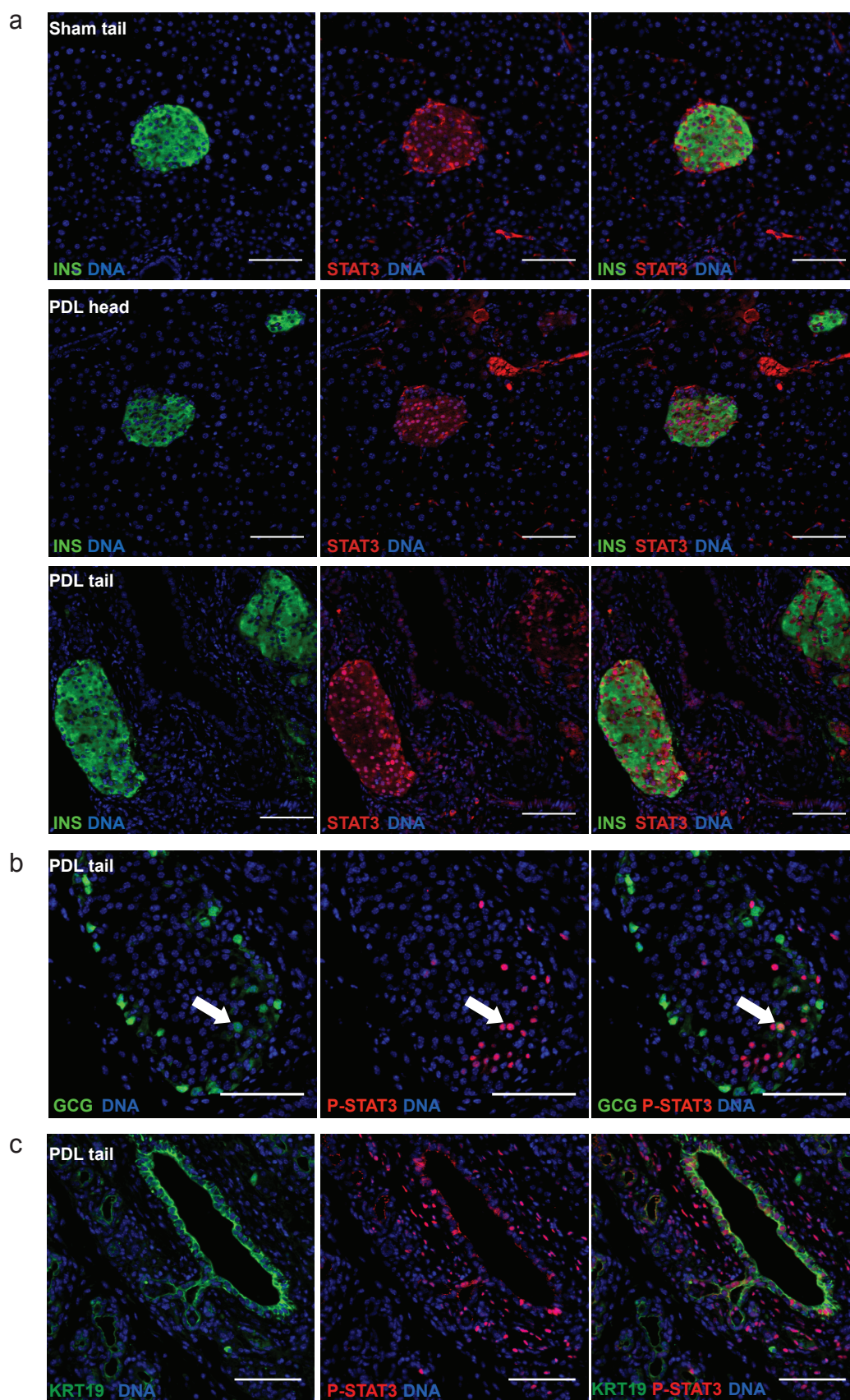

Figure S2

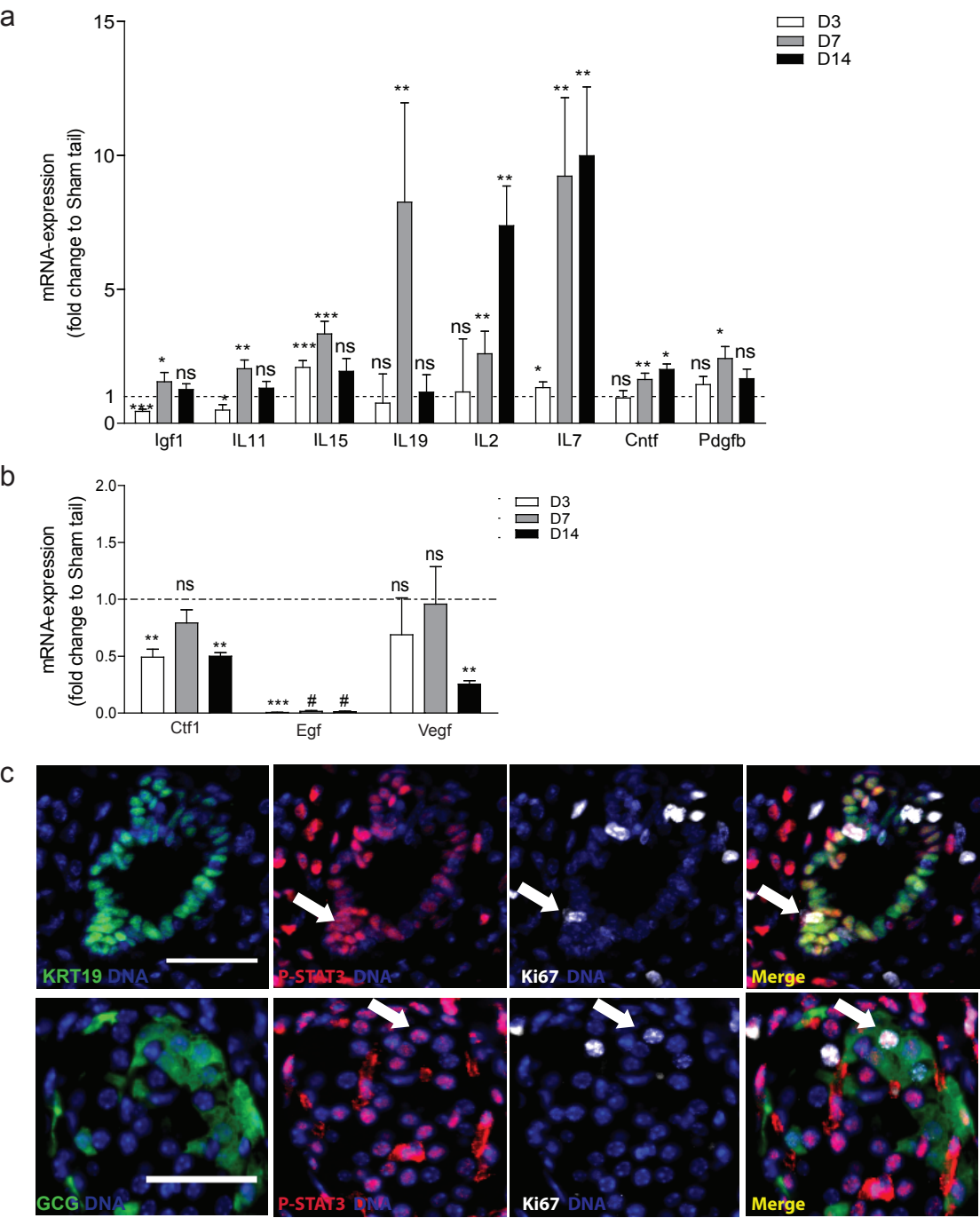

Figure S3

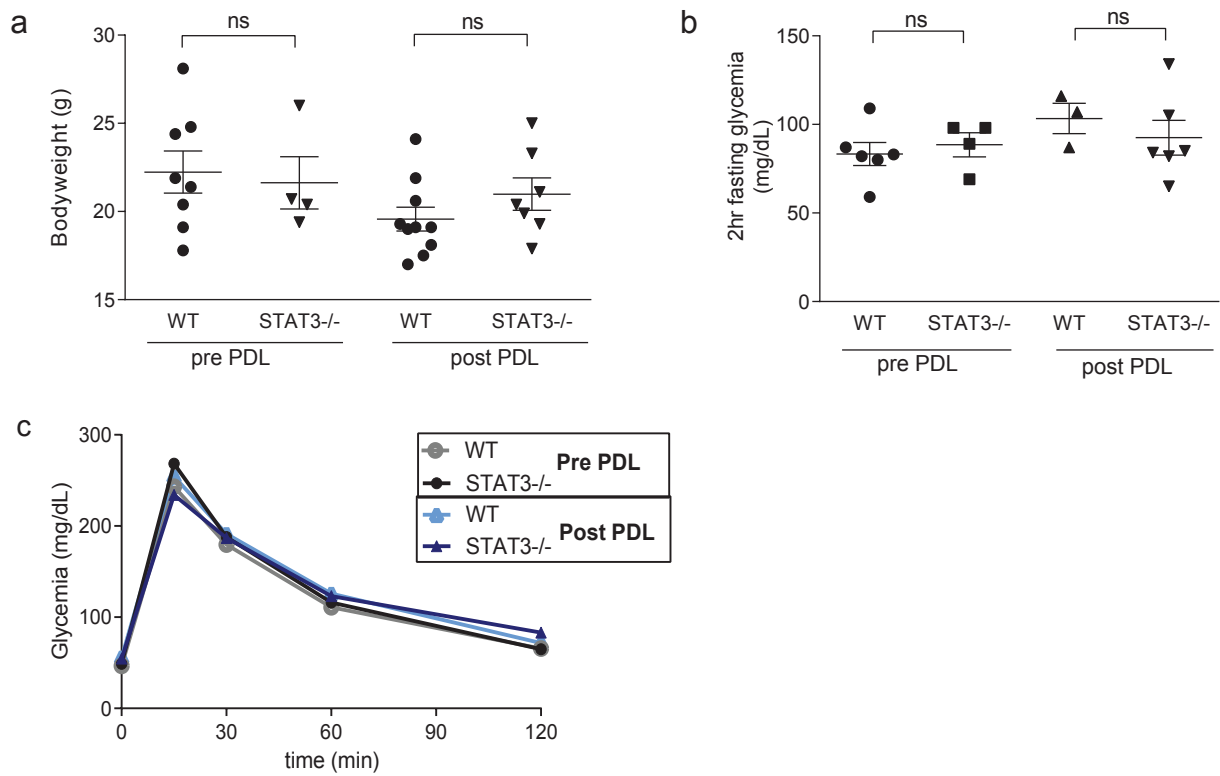

Figure S4

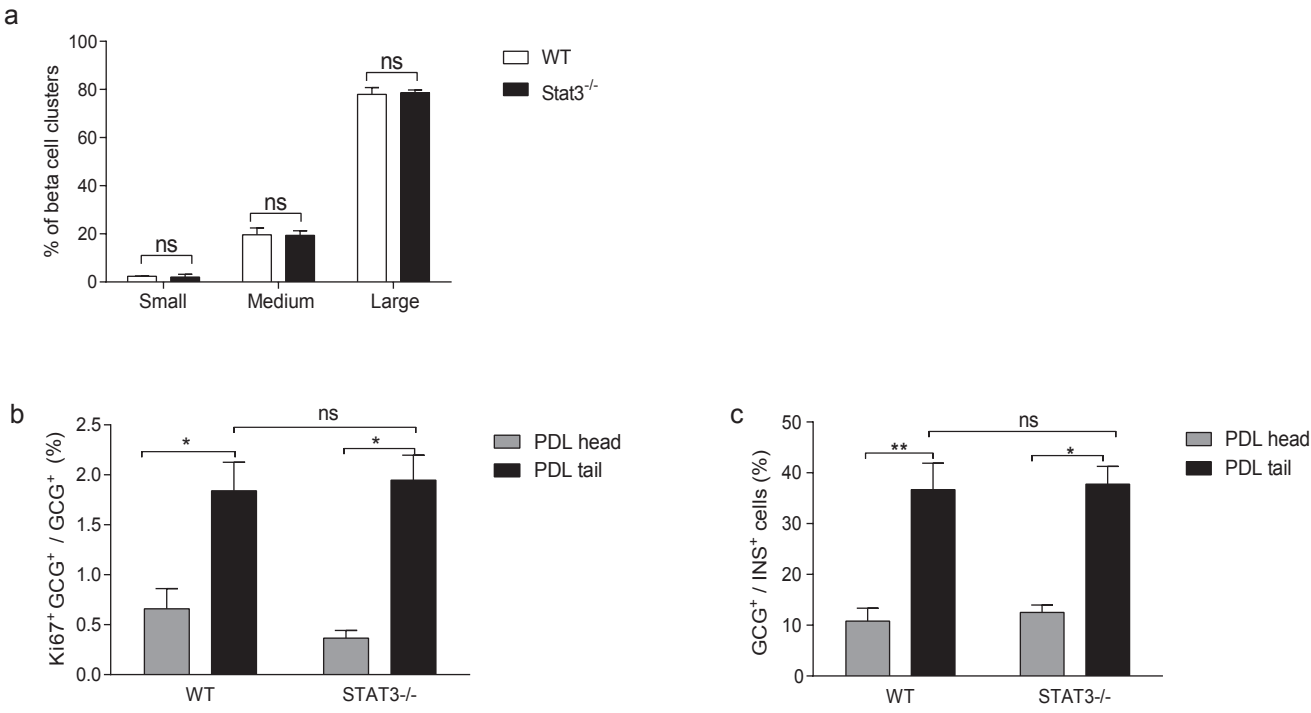

Figure S5

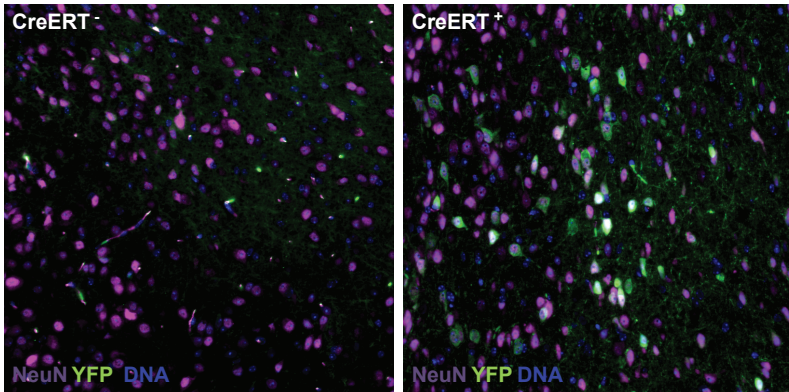

Supplement: Supplementary Information [file cddis2016171x1.pdf]
